# Assessing the Potential of Ancient Protein Sequences in the Study of Hominid Evolution

**DOI:** 10.1093/gbe/evag035

**Published:** 2026-02-27

**Authors:** Ioannis Patramanis, Laurits Skov, Enrico Cappellini, Fernando Racimo

**Affiliations:** Globe Institute, University of Copenhagen, Copenhagen, Denmark; Globe Institute, University of Copenhagen, Copenhagen, Denmark; Globe Institute, University of Copenhagen, Copenhagen, Denmark; Globe Institute, University of Copenhagen, Copenhagen, Denmark

**Keywords:** palaeoprotoemics, phylogenetics, population genetics, human evolution, incomplete lineage sorting

## Abstract

Palaeoproteomic data can provide invaluable insights into hominid evolution over long timescales. Yet, the potential and limitations of ancient protein sequences to resolve evolutionary relations between species remains largely unexplored. In this study, we aim to quantify how much information about these relations can be obtained from limited ancient protein data, at the scale that is currently available or will be available in the near future. We harness sequence alignments of 12 enamel and collagen proteins that have been previously reported in fossil material that is at least 1 million years old. We utilize in silico translations of hominid DNA sequences of these proteins and highlight their differential sequence conservation, indicating some of them contain much larger amounts of information than others. We also evaluate the extent to which inferred topologies from protein data differ from inferred topologies from the more informationally dense DNA data. We show that the former may sometimes lead to inferences of the wrong tree topology due to the informational loss that comes when working with peptide data. Additionally, we determine the number of concatenated proteins necessary to confidently reconstruct the population/species tree summarizing the relations between humans, chimpanzees, and gorillas, as well as those between modern humans, Neanderthals, and Denisovans. As expected, increasing the number of proteins in a concatenation enhances resolution, but we note that trees inferred from the full set of collagen and enamel proteins do not necessarily correspond to population trees inferred from genome-wide data. We show this is especially the case in the closely related groups of our recent ancestors. We further demonstrate that while a number of proteins fall within archaic introgressed haplotypes of present day humans, ancient admixture is not the main source of the observed tree incongruence. Our study underscores the potential and limitations of utilizing palaeoproteomic data in deep time phylogenetic reconstructions, indicating that these will be aided not only by increased recovery of proteins in the future, but also by more careful modeling of evolutionary relations across the genome, beyond simply building single phylogenetic trees.

Significance Ancient protein peptides can be recovered from fossils dating back to millions of years. These short sequences can offer us invaluable information on the evolutionary relations of extinct species. However, the accuracy and limitations of phylogenetic reconstructions based on this resource are still relatively unknown. Our work quantifies the information found within the enamel and type I collagen proteins, and shows that inferences based on protein data may sometimes differ from those of the DNA that translated them. We demonstrate that while the relationships between great apes can be easily resolved with a few proteins, those between human lineages are more problematic. This work showcases the limitations of using this invaluable resource to accurately reconstruct evolutionary relationships, especially those of recently diverged and admixed lineages.

## Introduction

Understanding the evolutionary relationships between extinct humans and other hominin groups is a fundamental question in paleoanthropology. This problem has largely been approached via methodologies derived from comparative morphology, phylogenetics and, more recently, the study of ancient DNA (Lockwood et al. 2004; Lalueza-Fox and Gilbert 2011; Villmoare 2018; Tattersall 2022). In the last decade, improvements in the extraction and sequencing of ancient peptides have provided researchers with yet another source of evidence to tackle this problem. Paleoproteomics is an emerging field that addresses the deep-time limitations of ancient DNA, which degrades faster than the peptides of specific proteins (Warinner et al. 2022). Thus, ancient peptide sequencing has enabled the study of evolutionary relationships between organisms that lived from tens of thousands (Nielsen-Marsh et al. 2009) to millions of years in the past (Demarchi et al. 2016; Paterson et al. 2025). It has also yielded valuable data in areas where DNA tends to be poorly preserved, due to humid and warm climates (Dalén et al. 2023). These include regions of the world such as southern Europe (Welker et al. 2020), southern Asia (Welker et al. 2019; Kubat et al. 2023), and Africa (Green et al. 2025; Madupe et al. 2025), which are rich in archaic hominid fossil material. Palaeoproteomics thus holds the potential to explore questions that were previously impossible to address using morphological or DNA data alone, including resolving the species or population identity of hundreds of fragmentary fossil specimens for which limited or no DNA sequences are available. Although the study of ancient proteins holds great promise, it also harbors limitations, caused by the number of proteins that can be retrieved from ancient material, and by the nature of protein data.

While some studies have managed to recover tens or even hundreds of proteins from relatively young samples (Cappellini et al. 2012; Orlando et al. 2013; Warinner et al. 2014; Hill et al. 2015; Sawafuji et al. 2017; Jersie-Christensen et al. 2018; Lanigan et al. 2020; Fu et al. 2025; Tsutaya et al. 2025), only collagen type I, enamel-specific proteins, and a few others have so far been retrieved from million years old mammalian fossil material (Rybczynski et al. 2013; Buckley et al. 2019; Welker et al. 2019; Madupe et al. 2025; Paterson et al. 2025). As only a handful of protein sequences are so far recoverable, only small amount of useful genetic information can be obtained from them. The sequences of these proteins are also not complete; degradation over time breaks down the original proteins into smaller and smaller peptides. After thousands of years, many peptides do not survive this process and are therefore unrecoverable, while peptides that do, can incorporate ambiguous amino acids due to postmortem chemical modifications (Warinner et al. 2022).

Even when fully preserved, proteins inherently contain less phylogenetic information than DNA (Weber et al. 2021). This is both due to the degenerate nature of the genetic code (Lagerkvist 1978), and to the fact that negative selection tends to act upon protein coding sequences more strongly than in other regions of the genome, reducing sequence variation (Eyre-Walker and Keightley 2007). Consequently, ortholog proteins of closely related species often show limited or no variation. Amino acid mutations may also occur in the same position multiple times in different lineages, due to functional molecular constraints (Zou and Zhang 2015), leading to molecular convergence or homoplasy (Mendes et al. 2016). This convergence can create the illusion of close phylogenetic affinities and has been observed in multiple taxa (Bazykin et al. 2007; Rokas and Carroll 2008; Parker et al. 2013; Foote et al. 2015; Goldstein et al. 2015; Zou and Zhang 2015). Furthermore, when only a few proteins sequences from a given species are available, it is difficult to establish whether observed variants are fixed or polymorphic within that taxon (Kubat et al. 2023; Madupe et al. 2025). All of the above reduce the utility of amino acid polymorphisms for reconstructing evolutionary relationships.

Moreover, small sets of ancient peptides can provide very limited information about the full ancestral recombination graph, ie the graph structure that describes the full genealogical relationships of a set of individual genomes (Griffiths and Marjoram 1996; Lewanski et al. 2024). Reconstructions based on these peptides tend to focus on individual gene trees, which in turn provide only partial knowledge about overall population relationships. This may be because gene trees are affected by incomplete lineage sorting (ILS) in ancestral populations (Maddison 1997; Hobolth et al. 2011), or because admixture events between populations may not be represented in such trees (Lohmueller et al. 2010). Both ILS and admixture are of concern in species or populations that are closely related to each other (Charlesworth et al. 2005; Pollard et al. 2006; Sousa and Hey 2013; Huerta-Sánchez et al. 2014; Mailund et al. 2014). In African great apes, for example, multiple studies have detected both high levels of ILS (amounting to up to 30% of the genome) (Hobolth et al. 2011; Scally et al. 2012; Kronenberg et al. 2018; Rivas-González et al. 2023), as well as possible past admixture episodes, in all three extant genera (Green et al. 2010; Kuhlwilm et al. 2019; Pawar et al. 2023; Galtier 2024).

Although most of these limitations have been previously acknowledged (Welker et al. 2020; Demarchi 2021; Taurozzi et al. 2024; Madupe et al. 2025), little quantitative work has been done to assess the potential and limitations of ancient protein data at resolving population relationships (Buckley and Wadsworth 2014; Froment et al. 2021; Codlin et al. 2025; Fong-Zazueta et al. 2025). In this study, we specifically focus on 12 collagen and enamel proteins and their ability to resolve evolutionary relationships between species. We focus on these proteins because they have been previously recovered from biological material older than a million years. We center our analysis on the Hominidae family and its well-studied genetic history, based on DNA evidence (Prado-Martinez et al. 2013; Wall 2013; Castellano and Munch 2020; Yousaf et al. 2021), and leverage the high availability of genomic and protein data that exists for all four of its extant genera and some extinct populations (Meyer et al. 2012; Prado-Martinez et al. 2013; Prüfer et al. 2014, 2017). For each of the 12 proteins, we use only a single isoform, labeled as “canonical” in Ensembl, as this is what has been previously recovered in ancient material. Lastly, we investigate whether the recovery of peptides from a richer proteome, such as that of dentin or bone, would enhance evolutionary resolution (and if so, how much).

First, we measure the entropy and evolutionary conservation rates of these 12 proteins, using hominid alignments, ranking them based on the amount of information they provide and comparing them to other known conserved proteins. We further use the entropy metric to measure the informational loss that occurs when comparing intron-containing DNA alignments, to exon and to protein alignments of the same genetic locus. We additionally evaluate how inferred topologies differ between these alignments of different data type.

**Fig. 1.**
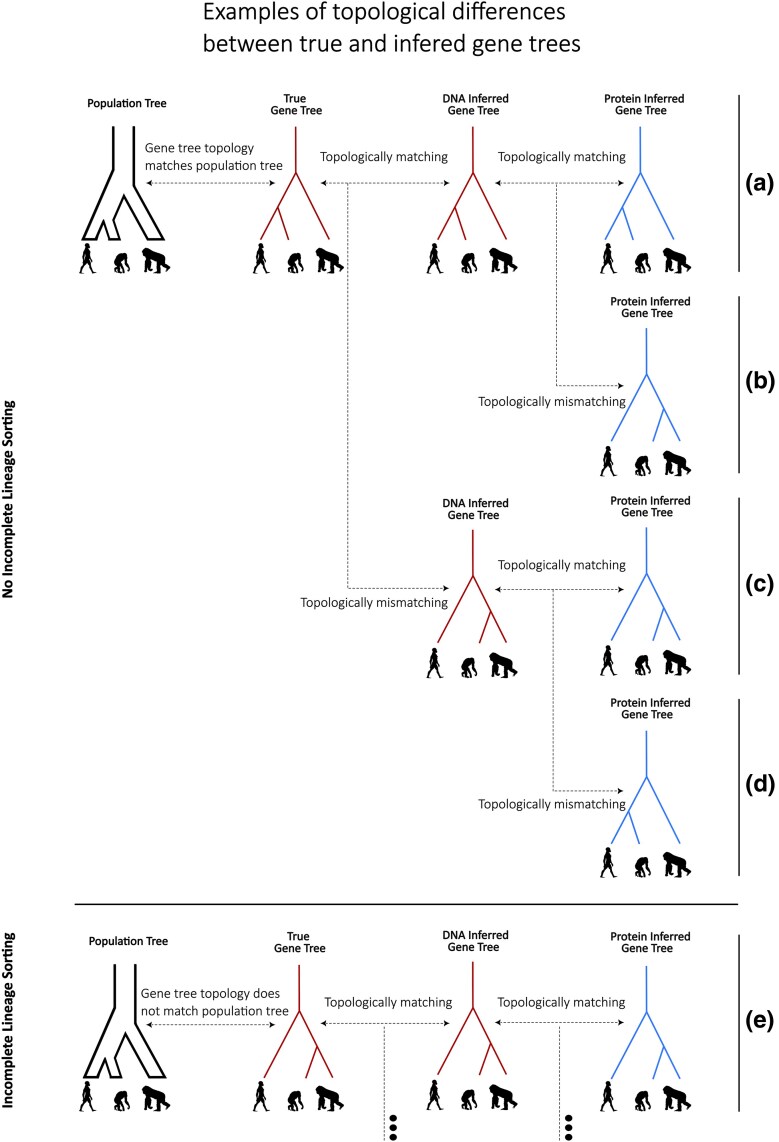
Schematic explaining different scenarios of tree topology discordance and concordance. The first column depicts the evolutionary relations between three different species (humans, gorillas, and chimpanzees), as described by a simple population tree. The second column depicts the tree representing the genetic relation between three homologous segments at a specific locus of genomes obtained from these three species (a “gene tree”). The third column depicts a tree inferred using DNA sequence data, obtained from said homologous region. The fourth column depicts the gene tree inferred using protein data from the same region. We show a (limited) set of the many possible scenarios that can arise when comparing all the above trees with one another: a) No ILS and all four trees agree with one another: the use of either DNA or protein data leads to the correct inference of the gene tree, which happens to agree with the population tree. b) No incomplete lineage sorting, but the protein gene tree differs from the true gene tree at the locus under study (eg due to the reduced information contained in peptide sequences, relative to DNA). c) No incomplete lineage sorting, but both the DNA- and protein-reconstructed gene trees are misinferred (eg due to very few genetic variants present at both the DNA and protein levels for correct tree resolution). d) No incomplete lineage sorting, but while the DNA-reconstructed gene tree is misinferred, the topology of the protein-reconstructed tree happens to match the true gene tree. e) There is actual incomplete lineage sorting (mismatch in topology between the true population tree and the true gene tree under study). In this specific case, the gene tree is also correctly inferred using either DNA or protein data. Other scenarios involving incomplete lineage sorting but misinferred topologies from either DNA data, protein data, or both have been omitted for brevity. All silhouette images are reused from https://www.phylopic.org/.

While ILS leads to topological mismatches between gene trees and the population tree, topological mismatches can also occur between the true and the inferred gene tree at a given locus, either when using DNA or protein sequences (see Fig. 1). DNA gene tree misinference can occur due to the inherent difficulties of reconstructing topologies from mutations, as well as from the information loss and errors that can take place during the sequencing process. Protein gene tree misinference, can occur due to the same reasons, but also the additional information loss that occurs in protein translation. Local tree reconstructions are also necessarily affected by difficulties in inferring recombination events and resulting topological changes along the genome. For simplicity in our empirical analysis, and given our focus on information loss in ancient peptides specifically, we assume that gene trees reconstructed from DNA sequences are accurate and use them as a proxy for the true gene trees. We also assume that the sequences we study are small enough to not be broken down by recombination events of the genome and that they can be characterized by a single gene tree. However, we note that both of these are strong assumptions that might not hold in reality.

We use an iterative analysis to compare protein-inferred phylogenetic trees with established tree topologies based on previously published genetic data (Meyer et al. 2012; Prado-Martinez et al. 2013). We estimate the number of combined proteins and amino acid variants required to reliably infer the population trees of three hominid genera and three hominin populations. We then repeat this iterative analysis by supplementing our 12 initial “deep time” proteins with 16 additional proteins, which have been experimentally recovered from bone or dentin (Rüther et al. 2022; Xia et al. 2024; Fagernäs et al. 2025), albeit so far, only from samples that are relatively younger. These 16 additional proteins have been previously ranked as the most abundant bone proteins and along with ALB, COL1A1, COL1A2, and COL2A1 are used in the SPIN species identification protocol (Rüther et al. 2022). We assess whether archaic introgression (interpopulation genetic contributions) is present on the genes coding these proteins and if it can influence the phylogenetic results. We additionally record the bootstrap support of our generated trees and investigate the relationship between this metric and the inference of incomplete lineage sorting on protein data.

## Results

### Incomplete Lineage Sorting, DNA, and Proteins

**Fig. 2.**
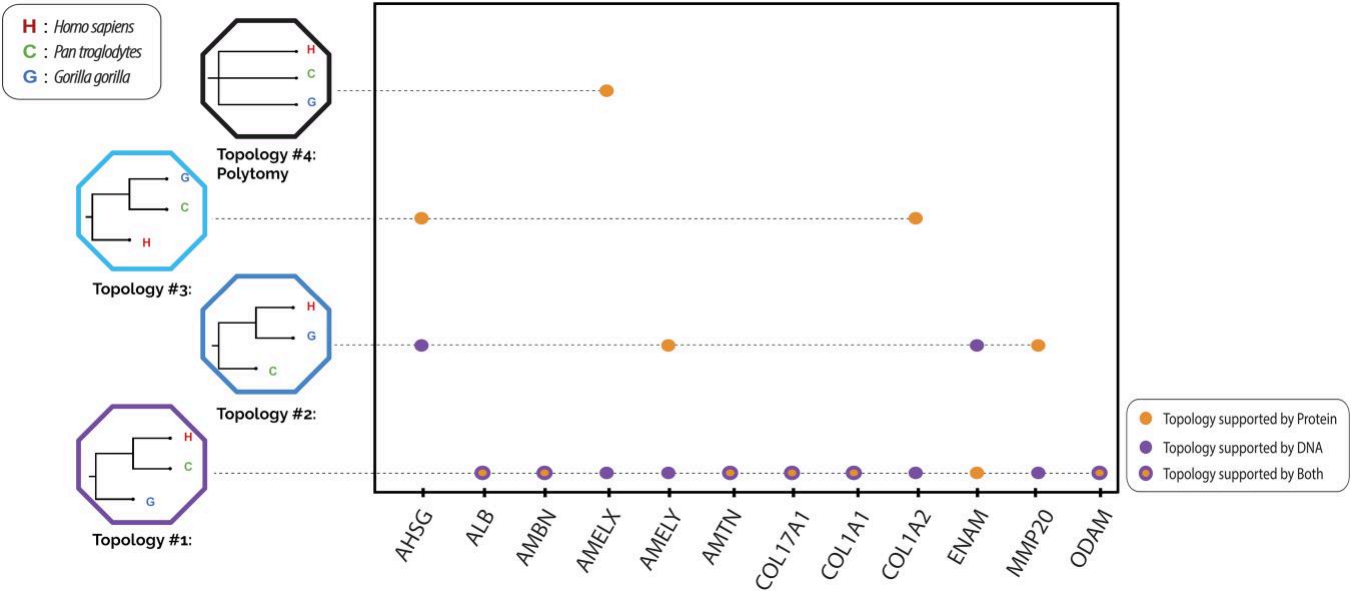
Comparison of topology supported by the 12 proteins under investigation and their corresponding DNA data. The four possible topologies are visible on the right side. For six loci, both DNA and protein data support the same topology.

We inferred gene trees from each of the 12 loci of interest and observed that different topologies were recovered, depending on which data type we utilized (Fig. 2). For the protein set, 5 out of the 12 gene trees displayed an estimated topology that was different from the population tree (as inferred from genome-wide data; Yousaf et al. 2021). In contrast, when utilizing the DNA sequences, corresponding to those 12 genes, only 2 out of the 12 gene trees differed in topology from the population tree. In all cases except two, when the protein data of a locus supported a topology different from the DNA data, the DNA data of that same locus supported the population tree topology. The two exceptions are: (i) AHSG, where both DNA and protein data supported an alternative topology, which also differed from each other and (ii) ENAM, where the DNA data supported an alternative topology, while the protein data supported the population tree topology.

### Entropy and Evolutionary Conservation Rates

**Fig. 3.**
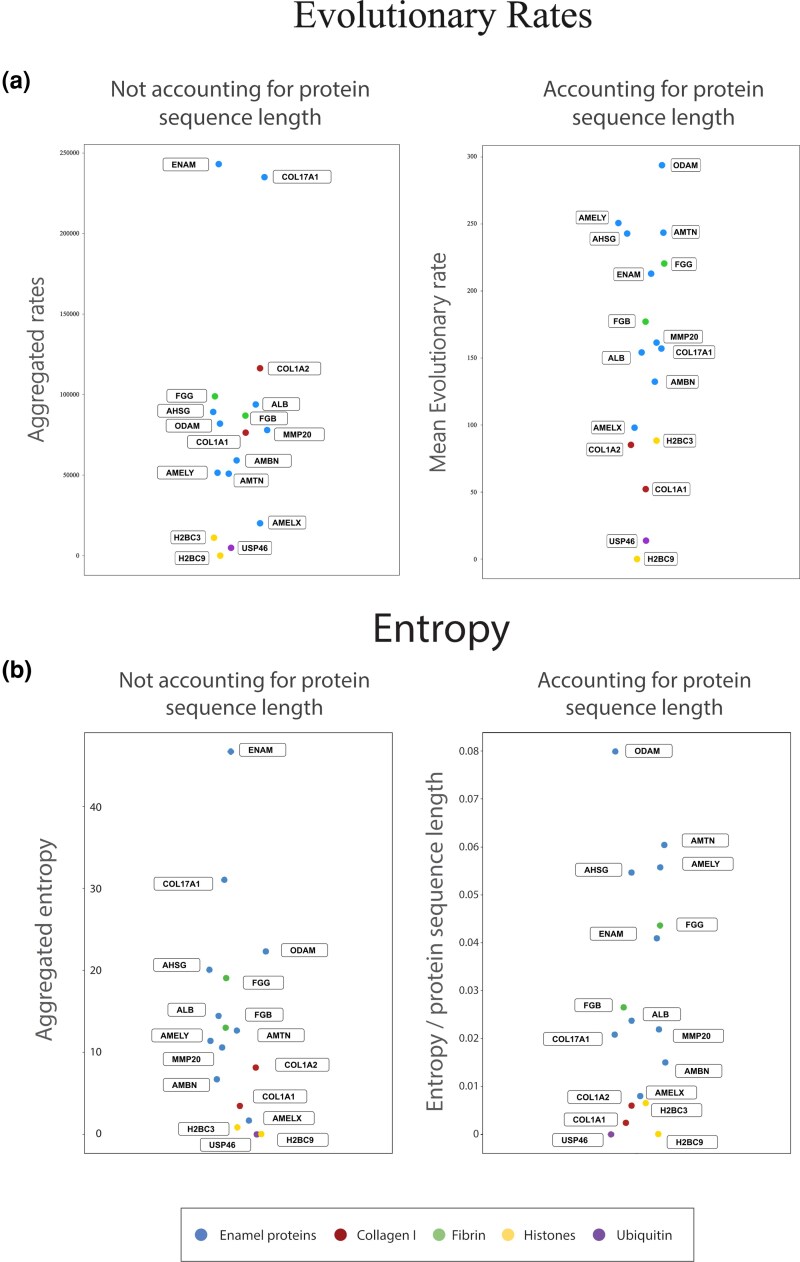
a) Protein entropy scoring comparison. Left: Each protein is ranked from highest to lowest based on the entropy scoring. Right: The entropy scoring is normalized based on the length of each protein, which causes some proteins to swap ranking. b) Protein evolutionary rates scoring comparison. Left: Each protein is ranked from highest to lowest based on the total evolutionary rate across all sites. Right: The total evolutionary rate is normalized based on the length of each protein, which causes some proteins to swap ranking.

We observe notable differences between the entropy levels and evolutionary rate scores of the proteins in question (Fig. 3). While the aggregated entropy and evolutionary rate rankings showed slight differences, when accounting for the length of each protein, both metrics showed a nearly identical arrangement of the proteins. When not accounting for protein length, ENAM, followed by COL17A1, were found to contain the highest amount of entropy as well as the highest evolutionary rate scores. When the entropy and evolutionary rate scores were divided by the length of each protein; however, ODAM was found to be the most variable, while proteins like AMELX, COL1A1, and COL1A2, fell on the lower end of the spectrum. When compared to known conserved proteins, almost all proteins showed substantially higher entropy and evolutionary rate scores than the ubiquitin and histone proteins that we compared them with, while the fibrin proteins fall within the range of the enamel proteins.

### Informational Content: Exons, Introns, and Proteins

When utilizing the entropy metric to examine the different data types, we observed that information decreased when comparing segments of mixed introns and exons to segments of pure exons and to those of amino acids (Fig. S1a and b). While the difference in entropy from the mixed intron and exon data to pure exons was substantial, the difference between the exon and the amino acid dataset was much smaller in comparison. When we normalized each entropy measurement by the length of the sequence that generated it, this drop in information content did not hold. In certain cases, the mixed intron and exon dataset contained less entropy than the pure exon dataset. In other cases, the amino acid alignment was the one with the highest entropy per site (Fig. S1c). Differences associated with each data type’s length (triplets of nucleotides corresponding to single amino acids) may be influencing these results. To correct this, we divided the average per-site information of the introns and exons and pure exons (seen in Fig. S1c) by 3, while keeping the protein measurement intact, in order to normalize this difference in datatype length. After applying this “length-correction” to the normalized entropies, we once again observe the original pattern of informational decrease for all genes (Fig. S1d), with the exception of AMELX. For AMELX, the pure exon dataset showed more information content than the mixed intron and exon dataset, even after the length-correction.

### Iterative Phylogenetic Analysis

**Fig. 4.**
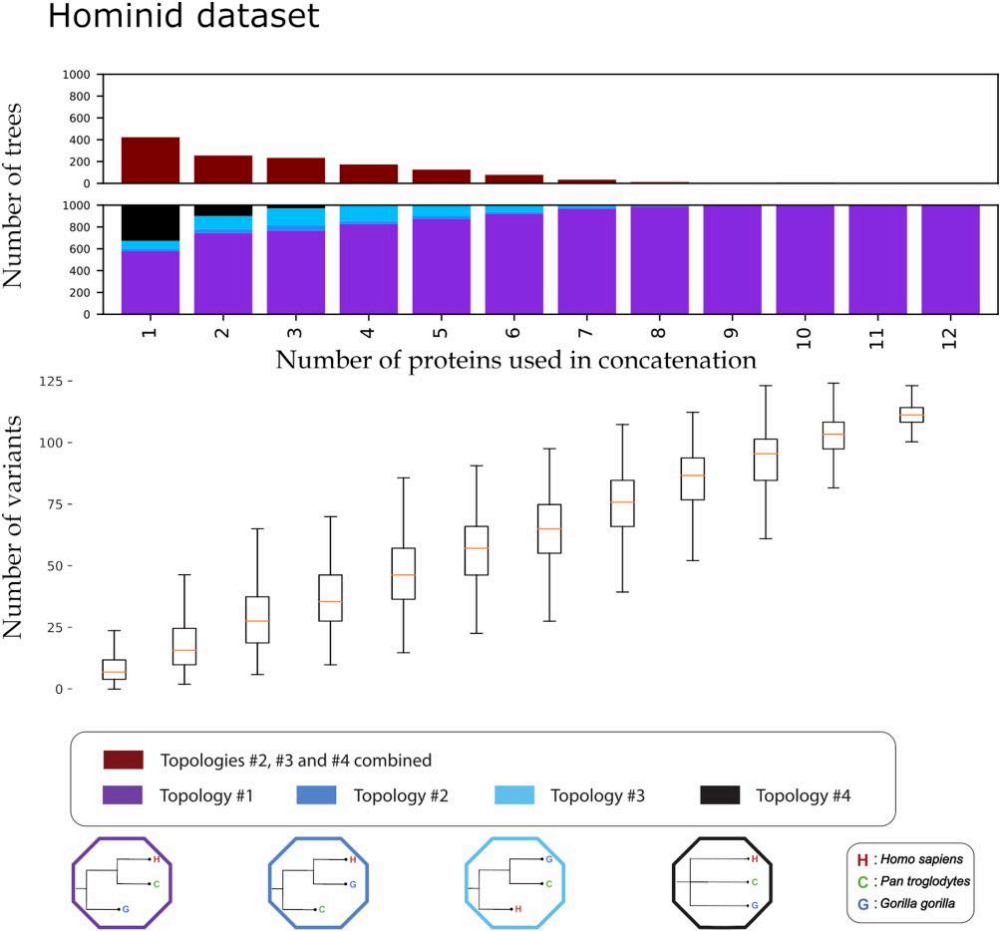
Iterative concatenation analysis for the hominid dataset. The upper barplot showcases the number of trees, out of 1,000, differing from topology #1 for an *N* number of proteins used in the concatenation. The lower barplot showcases the percentage of trees supporting each of the four possible topologies, out of 1,000, for an *N* number of proteins used in the concatenation. The four possible topologies and their corresponding colors are visible at the bottom of the plot. The number of variants present in the dataset creating each tree are visible below the bar plot as a box plot. For each box the orange line denotes the median of the variants present in the *N* number of proteins concatenation. Each box plot denotes the 25%, 50% (the median, the line in the middle of the box), and 75% quantiles of the distribution. The whiskers of each box denote extremely low values (25% − 1.5 * interquantile range) and extremely high values (75% quantile + 1.5 * interquantile range) for that distribution. The table containing the exact mean, median, maximum, and minimum variants for each *N* proteins is available in the Supplementary Material (S3.3).

Our consensus tree analysis yielded different results for the three different datasets it was applied to. For the hominid dataset (*Gorilla*, *Pan*, *Homo* using *Pongo* as outgroup), as the number of proteins and variants utilized in the concatenation increased, the number of consensus trees agreeing with the population tree topology (topology #1) increased as well, in an almost linear fashion (Fig. 4). At a lower number of concatenated proteins (*N* between 1 and 6), even if the majority of the consensus trees generated did agree with the population tree topology #1, large percentages of alternative topologies (#2, #3, #4) were also inferred. As an example, at N=1, 40% of the consensus trees had an estimated topology that was different from the population tree (though note here that the “consensus” is just a single gene tree for N=1). Topologies #2 and #3 were inferred in 20% of the iterations, with the other 20% corresponding to topology #4 (uninformative polytomy). This percentage of alternative topologies steadily decreased as *N* increased. At N=9, <5% of the consensus topologies differed from the population tree topology. When examining each of the discordant consensus topologies, unresolved polytomies between the three African great apes (topology #4) were represented at lower *N*s (N=1−3) but were absent at higher *N*s than 4. Above N=10, almost all consensus topologies converged into topology #1, the topology in agreement with the genome-wide DNA-inferred population tree. The number of informative sites (amino acid variants) that were used to generate each tree ranged from a mean of around 9 variants for 1 protein, to a mean of 113 variants for 12 proteins.

We further investigated whether the discrepancy in the amount of phylogenetic information between the enamel and collagen type I proteins can influence the generated tree topologies for the hominid dataset. To test this, we repeated the hominid tree analysis, this time excluding the two collagen type I proteins and using only the enamel specific ones. When doing so, we notice a slight increase in the trees in agreement with the known species tree, compared to the full enamel and collagen dataset (see Figs. S3 and S4). While no difference is visible when using between 1 and 5 proteins, there is a noticeable reduction in the number of discordant trees when using between 6 and 8 enamel-only proteins (or an increase in the trees supporting topology #1).

**Fig. 5.**
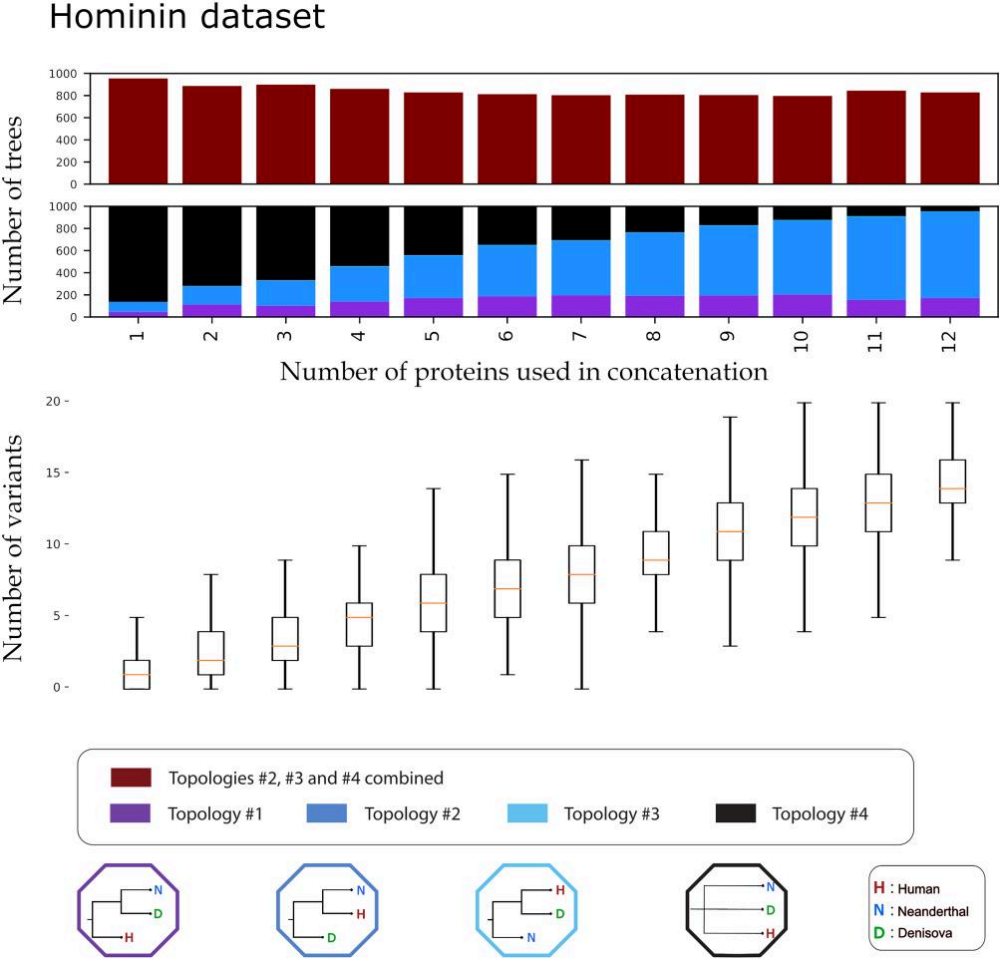
Iterative concatenation analysis for the hominin dataset. The lower barplot showcases the percentage of trees supporting each of the four possible topologies, out of 1,000, for an *N* number of proteins used in the concatenation. The four possible topologies and their corresponding colors are visible at the bottom of the plot. The number of variants present in the dataset creating each tree are visible below the bar plot as a box plot. For each box the orange line denotes the median of the variants present in the *N* number of proteins concatenation. Each box plot denotes the 25%, 50% (the median, the line in the middle of the box), and 75% quantiles of the distribution. The whiskers of each box denote extremely low values (25% quantile − 1.5 * interquantile range) and extremely high values (75% quantile + 1.5 * interquantile range) for that distribution. The table containing the exact mean, median, maximum, and minimum variants for each *N* proteins is available in the Supplementary Material (S3.3).

In contrast to the hominid dataset, increasing the number of proteins in the concatenation of the hominin dataset (modern humans, Neanderthals, and Denisovans using *Pan* as an outgroup) did not lead to an overall convergence to the genome-wide inferred population tree topology (topology #1) (Fig. 5). Instead, the number of trees supporting topology #1 remained roughly stagnant past the N=5 mark, while the trees supporting topology #2 (Neanderthal and modern humans as sister lineages) steadily increased. Furthermore at N=1, roughly 80% of the trees supported topology #4 (the polytomy). Although the percentage of topology #4 trees steadily dropped as the number of proteins increased, it did not completely disappear even with the use of 12 proteins (with 10% of iterations still leading to topology #4). For this dataset, the number of variants was roughly 10 times lower than that of the “hominid” and ranged from a mean of 1.2 variants for 1 protein, to 14.3 for 12 proteins.

**Fig. 6.**
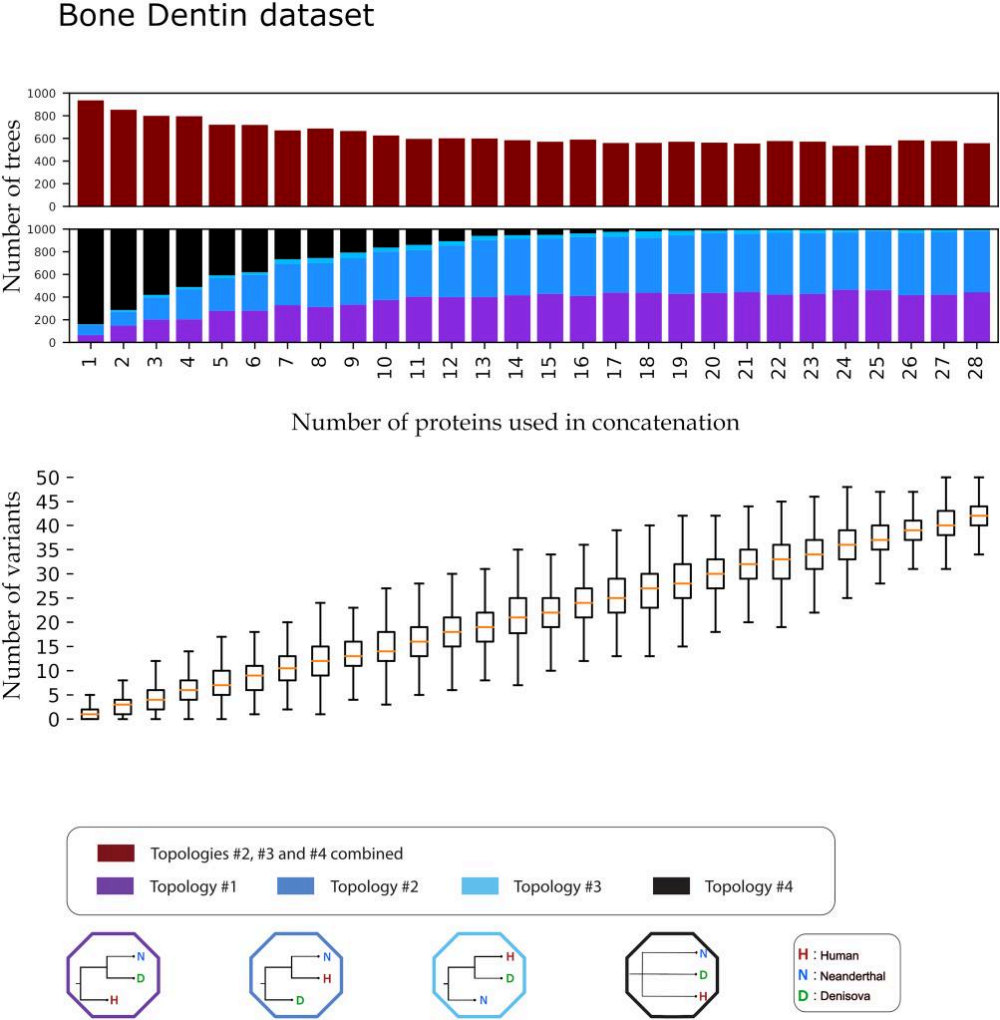
Iterative concatenation analysis for the dentin-bone dataset. The lower barplot showcases the percentage of trees supporting each of the four possible topologies, out of 1,000, for an *N* number of proteins used in the concatenation. The four possible topologies and their corresponding colors are visible at the bottom of the plot. The number of variants present in the dataset creating each tree are visible below the bar plot as a box plot. For each box, the orange line denotes the median of the variants present in the *N* number of proteins concatenation. Each box plot denotes the 25%, 50% (the median, the line in the middle of the box), and 75% quantiles of the distribution. The whiskers of each box denote extremely low values (25% quantile − 1.5 * interquantile range) and extremely high values (75% quantile + 1.5 * interquantile range) for that distribution. The table containing the exact mean, median, maximum, and minimum variants for each *N* proteins is available in Supplementary Material (S3.3).

When including additional proteins to the hominin dataset (ie the dentin-bone dataset), we observed that the greater set of available proteins decreased the proportion of trees supporting topology #2 and increased the proportion supporting topology #1 (Fig. 6). In this more protein-diverse dataset, a total of 12 proteins supported either of these two topologies (#1 and #2) with roughly equal representation. Further increases in the number of proteins did not appear to change this proportion, but we did observe a continual decrease of the cases where topology #4 (polytomy) was inferred. This polytomy effectively disappeared at around 20 proteins or a mean of roughly 30 variants. At 28 proteins, or roughly 40 variants, both topology #1 and topology #2 were still equally supported.

### Introgression

**Fig. 7.**
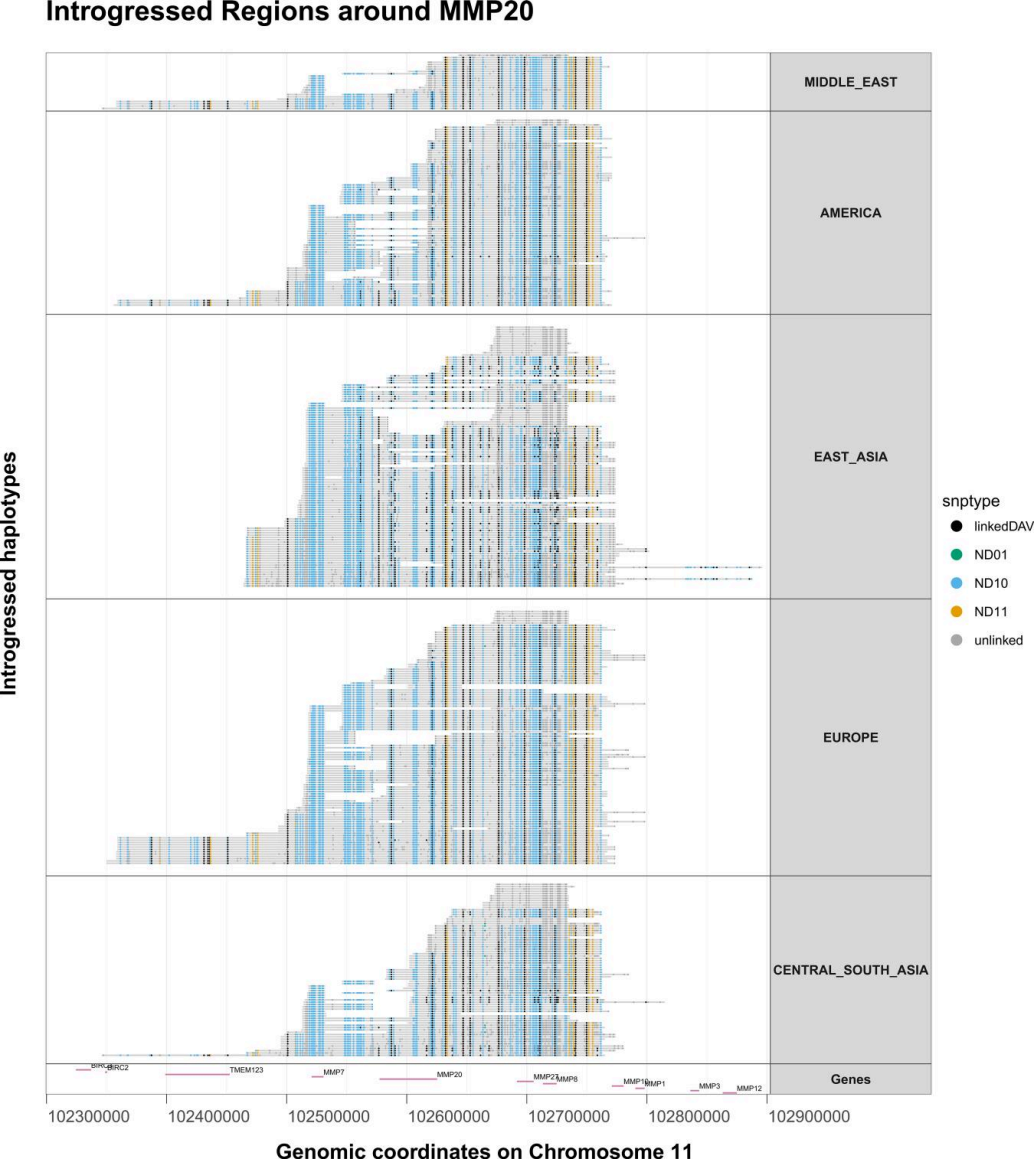
Introgressed segments (gray) overlapping the MMP20 gene and aligned to the human reference genome coordinates. Each row is an introgressed segment from a single chromosome of a specific individual. The segments have been grouped by continent and sorted by length. The dots on each segment represent single nucleotide polymorphisms (SNPs). These SNPs are either modern human derived polymorphisms that are linked (black) or unlinked (gray) to the introgressed segment, or introgressed archaic (orange), Neanderthal (blue) or Denisovan (green) polymorphisms. The last row showcases the range of the coding genes (n=11) corresponding to this region, centered around MMP20.

Our introgression investigation showed that some of the proteins investigated here are located within archaic-introgressed regions in present-day human genomes. The frequencies of the archaic variant of a protein can be very different between each protein and between various present-day human population panels (see Tables S5–S8 for the exact frequency results). In most cases, the observed frequency of the archaic-introgressed protein in the overall dataset is very low, <0.1%, but we found it to be very high in one of the 12 proteins: the archaic introgressed version of the enamel gene MMP20 has a frequency of around 18% in the global dataset but as high as 40% when looking at European and East Asian population panels alone. Plotting the introgressed haplotypes overlapping with MMP20 revealed a number of archaic tracks covering multiple related genes such as MMP7, MMP8, and MMP27, present in populations of almost every continent (Fig. 7). Other introgressed proteins showed a more localized introgression signal such as higher frequency for ALB and ODAM variants in Oceanian populations that match the Denisova 3 high coverage genome (see Table S7).

**Fig. 8.**
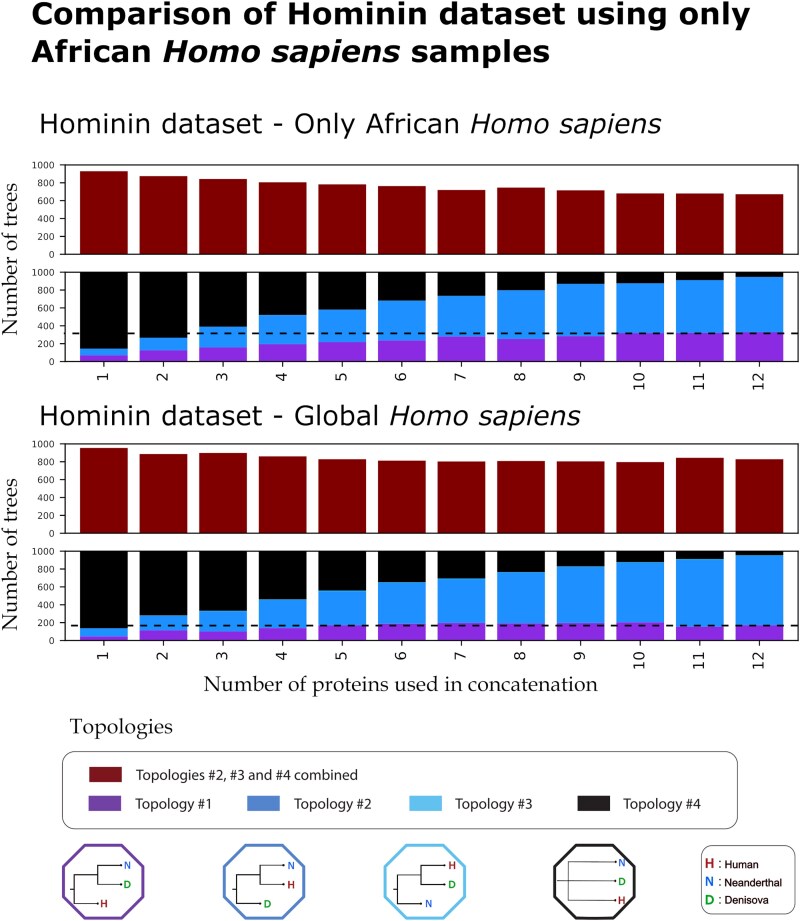
Comparison of iterative analysis results between using one of two methods for the hominin dataset a) only African samples as the *Homo sapiens* representative and b) a randomly chosen sample from any population of the 1,000 Genomes as the *H. sapiens* representative. For each of the two datasets, the first barplot at the top of the figure represents the number of trees, out of 1,000 repetitions, differing from topology #1, for each number of proteins used in a concatenation, ranging from 1 to 12. The second barplots breaks down the number of trees supporting each of the four topologies for the same number of proteins. A black dotted line has been added that denotes the number of trees supporting topology #1 when using the maximum number of available proteins for each of the two methods.

The modified iterative analysis of the hominin and dental-bone datasets, using present-day humans only from four African population panels, revealed slight but noticeable differences (Fig. 8 and Figs. S11–S13). In contrast with the original iterative analysis of the hominin dataset, when we only used African individuals from the 1,000 Genomes, we observed a small but steady decline in the number of trees disagreeing with Topology #1. This included the trees generated when using the maximum of 12 enamel proteins or 28 enamel, bone, and dentin proteins. Similarly, the number of trees agreeing with topology #1, while before remained stagnant past four proteins in the enamel dataset, now slightly increases continually until the 12 protein mark (Fig. 8). For the bone-dentin dataset, the results of using only African *Homo sapiens* had a smaller impact and once again the overall dataset supports topology #2 being as likely as topology #1. Another consequence of using only the African population panels is the complete disappearance of topology #3 (the one with humans and Denisovans closest), which may have been a consequence of removing samples from Asia harboring Denisovan ancestry (we note the 1000 Genomes Project used by our iterative analysis, has no representation of populations from Oceania, who tend to bear a considerable proportion of Denisovan ancestry).

### Phylogenetic Support Metrics

Our results indicate that a high bootstrap support is a metric indicative of the robustness of the underlying protein data in supporting a topology. This is based on the fact that a mean high bootstrap support (>90%) was generated only in cases where the different combinations of proteins in the alignment all consistently supported the same tree, which was also matching the reference population tree. Lower bootstrap supports (<50%) were associated with topologies that were under represented in the total number of trees, while medium bootstrap supports (75%<90%) were usually indicative of multiple topologies that were presented in equal numbers. The generated figures and a detailed review of the bootstrap results are available in the Supplementary Material (S3.5).

## Discussion

Palaeoproteomic data hold immense potential for the study of hominid evolution. Ancient proteins are already being used to track the distribution of different species through space and time (Brown et al. 2016; Chen et al. 2019; Brown et al. 2021; Demeter et al. 2022; Xia et al. 2024; Fagernäs et al. 2025; Fu et al. 2025; Tsutaya et al. 2025) and to assess the taxonomic placement of specimens (Welker et al. 2019, 2020; Kubat et al. 2023). Yet so far, only a few studies have attempted to investigate the phylogenetic potential of the various recoverable ancient proteins. Buckley and Wadsworth (2014) and Froment et al. (2021) have previously approached this question experimentally, by sequencing bone and tooth proteomes respectively, and identifying which proteins and peptides were recoverable from the investigated tissue. They used these recovered peptides to reconstruct phylogenetic trees of different taxa, inspecting those phylogenies for accuracy (by comparing the resulting topology with known species relations), confidence (by examining the bootstrap support of the trees), and resolution (by enumerating generated polytomies). The informativeness of those proteins was also assessed, by counting the number of species-informative variants. More recently, Fong-Zazueta et al. (2025) delved deeper into this question, using a combination of dry and wet-lab methodologies. In this work, missingness was introduced into in silico predicted enamel protein sequences, based on experimentally observed patterns of degradation. The in silico degraded sequence alignments were then used to generate phylogenetic trees, assessing their topological distance from a DNA-supported tree. Lastly, in a recent publication, Codlin et al. (2025) translated avian egg-shell and collagen type I proteins, assessing their phylogenetic resolution and accuracy, but also highlighting the existence and possible effects of within-taxon amino acid variability.

The above studies have demonstrated some of the potential—as well as the limitations—of using palaeoproteomic data for reconstructing evolutionary relations. Questions centered around the scarcity of data, as a result of postmortem degradation, have understandably stood at the forefront. Yet, the works described above have either focused on individual proteins or groups of proteins as a whole. In our work, we acknowledged the fact that researchers rarely have the option of choosing which proteins to utilize in their analysis, as this is usually simply the result of which peptides are recovered. As a result, we investigated the effect of incremental additions of protein data on the confidence and topology of the generated trees, exploring all possible numbers and combinations from a group of proteins. We also explored how well these protein sequences perform in reconstructing the relations of very closely related and recently admixed populations. While more challenging, these relations are often of great interest to evolutionary biology and to hominid evolution in particular. In our work, we only utilized in silico translated, complete amino acid sequences in order to simplify our analyses and comparisons with the (complete) available DNA data. We acknowledge this is never the case when working with real ancient protein data. To make the comparison more useful, we provide the number of variable amino acid positions used in each of our phylogenetic results, which can also be calculated on real data, and can be compared with the data presented here.

### Protein Informational Reduction and Incomplete Lineage Sorting

Multiple studies have previously identified high levels of ILS among great apes. For example, up to 30% of gene trees in a comparison between humans, chimpanzees, and gorillas have been shown to be under ILS (Hobolth et al. 2011; Scally et al. 2012; Kronenberg et al. 2018). Yet, the proportion of sequences under ILS drops significantly when investigating coding regions exclusively. Moreover, gene families displaying high levels of selection are even less likely to be undergoing ILS (Rivas-González et al. 2023). Of the 12 genes investigated here, only 2 of them (16%) showcased apparent ILS, as inferred from the DNA sequences (see Fig. 2). As the entropy and evolutionary rate results showcased, some of these genes are under high constraint due to selection, which may be the main driver for the relatively low level of observed ILS among them. However, when the corresponding proteins were investigated, 5 out of 12 (41%) led to the inference of a topology that was different from the population tree.

A likely explanation of this higher level of observed ILS when analyzing proteins is misestimation of the underlying gene trees, as a consequence of the reduced informational content of proteins relative to DNA. This “informational drop” between DNA and protein data types is supported by our entropy calculations, although admittedly, the question of “how much information” is contained within each data type, largely depends on how one measures said information: absolutely, per individual site or per individual codon (see Supplementary Material S2.1–S2.4). Removing the collagen type I proteins, two of the most conserved proteins of our dataset, led to a slight decrease in the number of discordant trees, when using more than five proteins. Similar results were also reported by Fong-Zazueta et al. (2025) who eliminated specific discordant trees by removing the conserved collagen proteins from their alignments.

These results suggest that inferences based on protein data alone, may lead to higher apparent levels of ILS than what is inferred when working with DNA data. Consequentially, protein-based phylogenetic inferences may distort evolutionary conclusions and overestimate the true amount of ILS present between taxons. Previous publications on modern proteomes have made similar arguments, based on the fact that closely related species showcase “apparent molecular convergence” due to inference errors from very conserved sequences (Mendes et al. 2016).

### Enamel and Collagen Conservation

Our combined entropy and evolutionary rate results indicate that the hominid enamel proteome consist of proteins of variable levels of conservation and information content (Fig. 3). All protein sequences, including collagen type I, appear more variable than the hyper-conserved ubiquitin and histone protein sequences that were used for comparison. When accounting for protein length, although some proteins like AMELX appear very conserved, all other enamel-related proteins yield greater average site variation, within hominids, than collagen type I proteins do. This pattern holds regardless of whether one measures variation through entropy or through evolutionary rate scores. As an example, ODAM displays more than 10 times the entropy score per amino acid than COL1A1 or COL1A2 and roughly three times their evolutionary rate score. These results are in line with the knowledge that collagen genes are heavily conserved in humans (Chan et al. 2008; Hudson et al. 2017). They also agree with Codlin et al. (2025), who showcased a higher conservation rate in avian collagen sequences compared to eggshell proteins, and Fong-Zazueta et al. (2025), who also noted the high conservation of collagens compared to enamel proteins in primates. A notable exception to this is AMELX: while being the most abundant enamel protein (Brookes et al. 1995), it shows a similarly conserved sequence to collagen type I. Yet, while AMELX tends to display low sequence variation, its Y chromosome isoform, AMELY, is much more variable, especially when accounting for its short length. This should not be unexpected: AMELY is located on the nonrecombining region of the Y chromosome where it is evolving under less selective constraint and under a faster local mutation rate (Hughes et al. 2010; Helgason et al. 2015; Sayres 2018).

Our work is an initial investigation into the informativeness of AMELY, a protein that has recently become of great interest (Stewart et al. 2017; Parker et al. 2019; Casas-Ferreira et al. 2022; Adair et al. 2024; Cleland et al. 2024; Gamble et al. 2024; Koenig et al. 2024) due to its ability to identify the biological sex of heavily degraded samples (Lugli et al. 2019; Froment et al. 2020; Cintas-Peña et al. 2023). Although it is expressed at lower concentration than AMELX (Cole and Eastoe 2014), AMELY can provide useful information for species identification and phylogenetic inferences, due to its high variability. However, analyses based on AMELY are also fraught with difficulties. For most vertebrate species, even when the sequence of AMELX is well characterized, the amino acid sequence of AMELY and the location of its coding gene are unknown (Martin et al. 2023; Ensemble homo sapiens amely orthologs n.d.). In some taxonomic groups, the gene responsible for expressing AMELY is missing in its entirety (Snead et al. 1989). Finally, as noted by Fong-Zazueta et al. (2025) (who for practical reasons chose not include AMELY in their enamel protein investigation), in some taxons the genes of AMELX and AMELY are not acting as independent loci (Janečka et al. 2018; Kawasaki et al. 2020), limiting their phylogenetic utility. As a result, while our analysis here showcases the high informativeness of AMELY—in contrast with that of AMELX—we recommend caution when working with this protein for evolutionary inferences.

Today, protein-based archeological species identification primarily relies on collagen (type I) mass fingerprinting (Warinner et al. 2022; Buckley 2023). Our results indicate that, as a whole, the enamel proteome evolves faster than collagen type I and thus, when the appropriate tooth tissue is available, could differentiate between more closely related populations or species. This is especially important given the microdestructive techniques, such as acid etching, that have successfully been applied to tooth enamel and bone material (Griffin et al. 2008; Stewart et al. 2016; Rebay-Salisbury et al. 2020; Fabrizi et al. 2024). These methods can extract useful amino acid sequence information using a minimal amount of material, inflicting only minor surface damage but preserving morphological information.

### Number of Proteins and Phylogenetic Resolution

The 12 proteins investigated here have previously been used in different combinations and have been shown to discriminate between the 4 extant genera of the hominidae family (Welker et al. 2019, 2020; Madupe et al. 2025). Nevertheless, the exact number of proteins required to reliably infer relations between these species is not yet clear. Our iterative analysis on the hominid dataset showed that, as one might expect, the number of consensus trees supporting an alternative topology to that of the population tree, drops significantly with the inclusion of additional proteins in the analysis. An increasing number of proteins sees an overall drop in discordant trees, in a linear decay, up to around 9 or 10 proteins, which we conclude to be sufficient for consistently recovering the reference population tree. Additionally, a combination of any two of the 12 proteins drops the percentage of polytomies from the population tree from 30% (when using only a single protein) down to <20%, and a combination of four proteins, to <1%. Thus, simply distinguishing between these three groups (without accurately inferring their phylogenetic relationships), should be possible with the recovery of around 4 of any of these proteins. African great apes have a rough estimated divergence time of between 5 and 9 million years (Castellano and Munch 2020). We expect other taxa with similar genetic distances to have similar phylogenetic resolution and power using palaeoproteomic data.

For our hominin dataset instead, our analysis revealed that the number of consensus trees agreeing with the topology of the population tree, did not increase with the inclusion of additional protein sequences in the analysis. Steadily increasing the number of these proteins from 4 to 12 showed little to no improvement in resolving this phylogeny. Instead, increasing the number of proteins from this set led to an increase in support for one of the three alternative topologies, topology #2, the one with modern humans and Neanderthals as sister lineages. Indeed, this one particular topology (itself discordant relative to the population tree) is supported by the fully concatenated 12-protein dataset we chose for this analysis (also shown previously by Welker et al. 2020).

The inclusion of additional proteins from the bone-dentin dataset led to slightly different results. Instead of a single topology that does not match the population tree becoming increasingly supported, two competing topologies (topology #1 and topology #2), equally represented, become the most commonly observed when using between 10 and 28 proteins. Similarly, a polytomy between these three groups was still present when using up to 20–24 proteins, although admittedly in extremely low frequency. Our analysis reveals that when examining very closely related populations with limited protein data, the addition of a few more protein sequences may not always increase support for the tree that is closest to the population tree of said taxons, as inferred from genome-wide data. In such cases, trees with medium to high bootstrap values (70%–80%) may be obscuring incongruence in the underlying data. Likewise, distinguishing between these groups, a process necessary for species or population identification, is also a difficult task, largely dependent on recovering some of the few informative sites that exist.

One explanation for the above results is that the recent admixture between these groups (Green et al. 2010; Meyer et al. 2012), has led to some present-day humans carrying Neanderthal or Denisovan haplotypes that overlap with the genes coding the proteins under investigation. Here, we showed that (i) some present-day humans do carry the archaic-introgressed version of the studied proteins, in frequencies which also differ among populations, (ii) that controlling for this introgression by using unadmixed present-day humans in the phylogeny does increase the proportions of the trees that are in agreement with the whole genome data, and (iii) even when controlling for archaic admixture, the concatenated protein phylogenetic trees result in different alternative topologies with equal support for these three groups.

Other forms of admixture could also be influencing these results. Previous publications have hypothesized about a deeply archaic introgression into Denisovans (Prüfer et al. 2017), which would make this population more different than the present-day human or Neanderthal lineage. Alternatively, an earlier introgression of ancient African, anatomically modern humans into Neanderthals (Kuhlwilm et al. 2016; Chen et al. 2020), would also bring these two groups closer to each other than to Denisovans. Both such admixtures could help explain why the protein data support the topology of present-day humans being closer to Neanderthals (see Figs. 5 and 6).

Another explanation to this issue is that the fairly recent split between these three groups, estimated by some to be around 400,000 to 600,000 years ago (Prüfer et al. 2017), does not allow for accurate phylogenetic inference using the phylogenetically conserved protein data. Given the slow evolutionary rate of protein sequences in general, it is possible that not enough time has passed for these sequences to sufficiently differentiate from one another. To put things in perspective, when comparing the number of variants present in the alignments of all 12 enamel proteins of hominids to that of hominins, the former is roughly 10 times higher than the later.

### Closing Remarks and Future Prospects

Currently, enamel and collagen type I proteins remain the only phylogenetically informative biomolecules that are recoverable for mammalian fossil taxa in deep time (samples that are more than 1 million years old). Although this unique resource is unparalleled in terms of preservation (Rybczynski et al. 2013; Paterson et al. 2025), its phylogenetic potential may be more limited than previously thought (Welker et al. 2020). In hominids, many studies have already noted a lack of resolution at finer taxonomic levels: unresolved polytomies generated using the enamel proteome have been identified inside the genus of *Pongo* (between the three extant species) (Welker et al. 2019; Kubat et al. 2023), the genus *Gorilla* (between the two extant species), as well as within the genus *Homo* (between present-day humans, Neanderthals and Denisovans) (Nielsen-Marsh et al. 2009; Welker et al. 2020; Demeter et al. 2022). Polytomies have also been observed at a subspecies level, such as the divisions between subspecies of *Gorilla gorilla* and of *Pan troglodytes*. Nevertheless, the two species of the genus *Pan* (*P. troglodytes* and *P. paniscus*) can be confidently distinguished from one another using a concatenation of enamel proteins (Welker et al. 2019, 2020). The reason why some evolutionary relationships are easier to resolve than others needs to be further investigated, but probable causes include differences in split times, differences in the effective population size of ancestral populations and different levels of postdivergence migration (Mailund et al. 2014).

Both results from previous publications and the present study suggest that phylogenetic analyses of archaic hominid taxa based on palaeoproteomic data should be taken with a degree of caution. Overall, protein data may lead to higher amounts of gene tree misestimation, as a result of the data type used for tree estimation. Here, we have shown that the number of currently recoverable, deep-time proteins allows for the reconstruction of species relations at the level of genera in the hominid clade (divergence time between 5 and 9 million years). This is very encouraging, given this particular clade’s genetic history of recent splits, high levels of ILS (Hobolth et al. 2011; Scally et al. 2012; Kronenberg et al. 2018; Rivas-González et al. 2023) and past admixture events (Green et al. 2010; Kuhlwilm et al. 2019; Pawar et al. 2023; Galtier 2024). However, our results also indicate that the same data have limited power to resolve the population trees of more closely related groups, such as those within the hominin clade. Given these results, past palaeoproteomic conclusions concerning distinct genera, like the assignment of *Gigantopithecus blacki* (Welker et al. 2019) to the ponginae lineage, can be considered more robust. On the other hand, results concerning more closely related taxa, within the same genus, such as the assignment of *Homo antecessor* (Welker et al. 2020) as an outgroup to all late Pleistocene humans or possible substructure within *Paranthropus robustus* (Madupe et al. 2025), should be viewed with a degree of caution.

The issues described here are neither new nor unique to the field of palaeoproteomics. During the early decades of the field of molecular phylogenetics, the limited amount of sequence data at the time, initially proteins, and later on short DNA sequences, offered limited resolution when resolving clades of closely related species. As an example, early studies were unable to resolve the polytomy of the human, chimpanzee, and gorilla lineages (Sarich and Wilson 1967) and identify which species was our closest living relative. This issue was not resolved until the accumulation of sufficient data roughly two decades later (Holmquist et al. 1988). Similarly, early ancient DNA studies based only on mitochondrial DNA, supported a scenario of “no admixture” between Neanderthals and modern humans (Currat and Excoffier 2004; Hodgson and Disotell 2008). The first published mitochondrial DNA from a Denisovan, characterized them as an outgroup to Neanderthals and modern humans (Krause et al. 2010). Once again, these relationships were reconsidered and resolved with the acquisition of more molecular data.

Increases in overall ancient peptide acquisition through novel lab methodologies (Jensen et al. 2023; Fagernäs et al. 2024, 2025; Wilkin et al. 2024) may lead to more confident phylogenetic placements and enhanced evolutionary resolution for these taxa. Studies extracting the bone proteome of younger samples have so far delivered a greater number of proteins (Cappellini et al. 2012; Orlando et al. 2013; Hill et al. 2015; Sawafuji et al. 2017; Lanigan et al. 2020). As an example, two recent publications have managed to recover an impressive amount of bone and dental proteins (n=51  Tsutaya et al. 2025, n=88  Fu et al. 2025), which they used to phylogenetically assign two fossil specimens to the Denisova clade, expanding our understanding of this enigmatic group. Future advances, such as targeted proteomic approaches (Gallien et al. 2012; Koenig et al. 2024), the identification of better preserving bones (Ásmundsdóttir et al. 2024) or improvements in downstream spectra identification (Chiang et al. 2024; Rodriguez Palomo et al. 2024) may allow for similar recoveries in older samples. However, we believe that an even greater number of proteins than the ones investigated here (n=28) will be necessary for the accurate resolution of evolutionary relations for very closely related populations or species. Alternatively, more computationally intensive methods of inference beyond concatenation, such as the multispecies coalescent, could result in better resolution by better accounting for the evolutionary processes that lead to different gene trees along a sequence (Lanier et al. 2014; Jiao et al. 2021). Given that the protein data examined here features high incongruence and a low number of informative loci, it is possible that tools like *Beast (Douglas et al. 2022) might provide results that better agree with the evolutionary relationships inferred from DNA.

On top of this, the population tree itself may be a poor representation of the overall relationships between closely related groups, due to admixture events (Green et al. 2010; Meyer et al. 2012; Prüfer et al. 2014, 2017). Indeed, when investigating the relationships between organisms that are as closely related as the ones investigated here, concepts such as “species” or “trees” lose some of their utility. A growing body of work from the field of ancient population genetics has shown that admixture between even distantly related groups of hominids might be the standard rather than the exception (Martin and Jiggins 2017; Kuhlwilm et al. 2019; Pawar et al. 2023). This seems to be especially true within the confines of hominin evolution during the Late Pleistocene (Wall and Hammer 2006; Lalueza-Fox and Gilbert 2011; Dannemann and Racimo 2018; Ackermann et al. 2019). In light of these discoveries, the field of paleoanthropology is also changing. Past quests for a single population tree are now slowly being replaced by the concept of a “braided stream,” a network of reticulating lineages that can split as much as they can merge (Ceder 2018; Ackermann et al. 2019; Tattersall 2022). Reconstruction of hominin evolutionary relations using other topological objects beyond trees (like admixture graphs) is still in its infancy, and so far, nonexistent in the field of paleoproteomics. This, in turn, suggests a fruitful avenue for future methodological developments.

## Materials and Methods

### Incomplete Lineage Sorting, DNA, and Proteins

We first selected 12 proteins that have previously been recovered from either tooth enamel (AHSG, ALB, AMBN, AMELX, AMELY, AMTN, COL17A1, ENAM, MMP20, and ODAM) or bone material (COL1A1 and COL1A2), from mammalian samples that are more than 1 million years old (Rybczynski et al. 2013; Buckley et al. 2019; Welker et al. 2019; Madupe et al. 2025; Paterson et al. 2025). We then acquired the “canonical” isoform’s reference sequence for each of the proteins from Ensembl (Martin et al. 2023), for the following four hominid species: *H. sapiens*, *P. troglodytes*, *Gorilla gorilla*, and *Pongo abelii*. We aligned the ortholog sequences from the four hominid species using Mafft (Katoh and Standley 2013) and reconstructed gene trees with PhyML (Guindon et al. 2010), using each of the 12 ortholog alignments separately. We rooted the 12 generated trees using *Pongo abelii* as the outgroup and compared them to the population tree that best represents the relationships between those four species (Wall 2013). To compare our protein tree results, we repeated the same process but using the reference DNA sequences (combined exons and introns) of the gen es corresponding to the 12 proteins instead of the amino acid sequences.

### Entropy and Evolutionary Conservation Rates

To assess the conservation levels and the phylogenetic information of these 12 proteins, we calculated Shannon’s information theoretic entropy (Shannon 2001) and an evolutionary rate score (Pupko et al. 2002) for each amino acid position on the sequence alignments. We used these two scores as approximations to the information content in these alignments, while respectively ignoring and accounting for the evolutionary distance between each sequence. We used Bio3d (Grant et al. 2006) for the entropy calculation and Rate4Site (Pupko et al. 2002) for the evolutionary rate computation on the alignments of the four hominid species. We obtained a score for each position of each of the 12 protein alignments. We aggregated the metrics across each protein to obtain a total score, and also divided them by the length of the alignment, to obtain a sequence-wide average score. To account for within-species diversity, we used multiple individuals as representatives from each of the four species, using previously published translated proteomes (Patramanis et al. 2023). We randomly sampled a single individual from each of the four taxa (*Pongo*, *Gorilla*, *Pan*, and *Homo*) and calculated the entropy and evolutionary rates between them. We then repeated this random sampling for 1,000 repetitions, each time calculating the entropy and evolutionary rate between a different set of four individuals from these four taxa. We then calculated and reported the mean across these 1,000 repetitions. To contextualize our results, we selected five proteins that have been previously reported as being either highly conserved or as containing hyper-variable segments (Nei 1987) and included them in the entropy and evolutionary rate calculations. These included two highly conserved histones (H2BC3 and H2BC9) and one ubiquitine (USP46) (Schlesinger and Goldstein 1975; Baxevanis and Landsman 1996), as well as two fibrinogen proteins (FGB and FGG) reportedly bearing a highly variable segment (Nei 1987).

### Informational Content: Exons, Introns, and Proteins

We assessed the differences in informational content between a DNA sequence containing both introns and exons, a version of the same sequence containing only exons, and a peptide version of the sequence (translated amino acids) for all 12 loci of our analysis. We applied Bio3d’s entropy scoring to all three data types of the same gene, for all 12 genes, and then ranked the results (see Supplementary material for details). We also divided each entropy metric by the length of the data type to compare the average information content per site, of each data type. Due to sequence length differences between DNA and protein data (with a three letter DNA codon corresponding to a single protein amino acid), we also applied a “length-correction” to this last measurement. In this correction, we divided the entropy of each protein version of each gene by three (number of nucleotides that correspond to an amino acid), while keeping the introns-and-exons and exons-only version unaltered.

### Iterative Phylogenetic Analysis

We investigated how the concatenation of different numbers and different combinations of the 12 proteins might affect the topology of the inferred “consensus” tree, which is often taken as an estimate of the population or species tree. For this analysis, we utilized a “hominid dataset,” consisting of *H. sapiens*, *P. troglodytes*, *Gorilla gorilla*, and *Pongo abelii* (as an outgroup) and a second “hominin dataset” consisting of *H. sapiens*, Neanderthals, Denisovans, and *P. troglodytes* (outgroup). To assess how the recovery of additional proteins, from different tissues affects phylogenetic analyses, we expanded the “hominin dataset,” creating a third “bone-dentin dataset”. This dataset consisted in the protein sequences that are most often recovered from dentin or bone tissue. In choosing which proteins to include in this analysis, we utilized the list provided by Rüther et al. (2022), which includes 20 proteins utilized in species identification: COL1A1, COL1A2, COL2A1, COL3A1, COL4A4, AHSG, COL5A2, ALB, BGN, COL5A3, COL5A1, CHAD, COL22A1, COL11A2, SERPINF1, F2, COL11A1, LUM, COL12A1, and POSTN. Four of these 20 proteins (COL1A1, COL1A2, AHSG, and ALB) were already included in the original 12 proteins, leading to a final combined dataset of 28 proteins.

In each iteration of this analysis, we carry out a concatenation using a subset of proteins sampled from the full set of proteins, reflecting the fact that not all proteins in the full set might be available in practice. The subset ranges in size from 1 (a single protein recovered) to all proteins recovered (either 12 or 28, depending on the tested dataset). One representative individual per population or species is randomly chosen and included in the alignment, as ancient protein studies are often limited to single individuals that are made to represent an entire species. For each concatenation, we build a phylogenetic tree and record the resulting topology. We then compare it to the underlying population tree, as inferred from past DNA studies. In total, we do this over 1,000 iterations per each number of proteins, sampling different sets of proteins and different representative individuals, in each turn.

We performed the same iterative analysis on each of the three datasets (“hominid,” “hominin,” and “bone-dentin”). The analysis for the “hominid” and “hominin” datasets was repeated 1,000 times for each *N*, with *N* ranging from 1 to 12, resulting in a total of 12,000 generated trees for each of the two datasets. The same process was applied to the bone-dentin dataset, with *N* ranging from 1 to 28, resulting in a total of 28,000 trees. For each iteration, we first picked *N* proteins, without replacement, out of the maximum number of proteins for that dataset. For each protein, one sequence from each of the four taxa was randomly selected from the samples available to us (Patramanis et al. 2022) and then the four orthologous sequences were aligned using Mafft. Each iteration (out of a thousand) thus generated a total of *N* protein alignments. The *N* alignments were then concatenated into a single alignment which was used to generate a phylogenetic tree using PhyML. The generated tree was also trimmed for very short and unsupported branches (see Supplemantary Material), which were transformed into polytomies. The tree was then rooted using an outgroup taxon (*Pongo* for hominid set, *Pan* for hominin, and bone-dentin set) and compared to a model reference tree. The model reference tree is a simple four-leaf tree that best describes the relations between the four taxa (Disotell 2012; Yousaf et al. 2021). In the case of the hominid set, the reference tree has the *Pan* and *Homo* nodes as the most closely related, followed by *Gorilla* as an outgroup to *Homo*-*Pan*. For the hominin set, the Neanderthal and Denisovan are the most closely related sister groups, with *H. sapiens* as the outgroup to the Neanderthal-Denisovan clade.

For each comparison of a generated tree with the model reference tree, we assigned a label (“Topology #1,” “Topology #2,” “Topology #3,” and “Topology #4”), each corresponding to one of the four possible topologies (including a polytomy). For both sets of taxons, the four topologies and their matching labels are shown in Figs. 4 and 5. We recorded the bootstrap support value of the node with the two most closely related taxons of the generated tree, excluding polytomies. Additionally, we enumerated the number of variant sites in the concatenated alignment (excluding informative sites of the outgroup taxon) that were used to generate each tree. All protein sequences for the iterative analyses were acquired from the “Hominid Palaeoproteomic Reference Dataset,” available on Zenodo (Patramanis et al. 2022). All alignments, concatenation and phylogenetic trees were generated using Module 2 of PaleoProPhyler (Patramanis et al. 2023). All downstream comparisons after generating the trees were done using scripts deposited on Github (see Supplementary material).

### Introgression

We further assessed the impact of admixture, as a contributor to apparent tree discordance. For this, we utilized the hominin dataset, given the known history of recent introgressions between the modern human, Neanderthal, and Denisovan lineages (Green et al. 2010; Meyer et al. 2012). We first identified how often the proteins under investigation here can be found within archaic-introgressed regions of present-day human genomes. We used previously reported archaic haplotypes found within two present-day human datasets (Skov et al. 2018; Chen et al. 2020) to assess this. The details of our methodology can be found in the Supplementary Material (S4). We also repeated the iterative analysis for the hominin and dental-bone datasets, to assess the effect of using largely unadmixed individuals when generating the phylogenetic trees. For this, we selected only individuals from the present-day human panels of the 1,000 Genomes: Yoruba, Mende, Luhya, and Mandinka, as the human representative. Previous studies have shown that these populations have reportedly the lowest amount of archaic introgression from the Neanderthal and Denisovan populations (Skov et al. 2018; Chen et al. 2020).

### Phylogenetic Support Metrics

To better understand the relationship between the results of our analysis and the confidence metrics generated by the phylogenetic software itself, we extracted and plotted the bootstrap support of each tree from all iterative analysis datasets. We grouped the bootstrap support scores according to the data set, the number of proteins used to generate them, and the tree topology they supported. We then plotted them as boxplots using python’s 3 Matplotlib (Hunter 2007) package, selecting the option to not plot outliers for visual clarity.

## Supplementary Material

evag035_Supplementary_Data

## Data Availability

The data and scripts to reproduce the entropy calculations between the different proteins and other data types (exons-only, exons-and-introns), along with the script to reproduce the results of Fig. 2, are available on Zenodo: https://zenodo.org/records/17530636 (Patramanis 2025b). The scripts and data, and download links to reproduce the iterative tree analysis, as well as the introgression investigation are available on Github: https://github.com/johnpatramanis/Protein_ILS_Hominids_and_Hominins (Patramanis 2025a).
